# Serum metabolomics characteristics and fatty-acid-related mechanism of cirrhosis with histological response in chronic hepatitis B

**DOI:** 10.3389/fphar.2023.1329266

**Published:** 2023-12-21

**Authors:** Hai-Na Fan, Zhi-Min Zhao, Kai Huang, Xiao-Ning Wang, Yun-Kai Dai, Cheng-Hai Liu

**Affiliations:** ^1^ Institute of Liver Diseases, Shuguang Hospital, Shanghai University of Traditional Chinese Medicine, Shanghai, China; ^2^ Shanghai Key Laboratory of Traditional Chinese Clinical Medicine, Shuguang Hospital, Shanghai, China; ^3^ Institute of Interdisciplinary Science, Shanghai University of Traditional Chinese Medicine, Shanghai, China

**Keywords:** metabolomics, PPAR γ, HBV-related cirrhosis, regression, fatty acids

## Abstract

**Background and aims:** The serum metabolites changes in patients with hepatitis B virus (HBV)-related cirrhosis as progression. Peroxisome proliferator-activated receptor gamma (PPARγ) is closely related to lipid metabolism in cirrhotic liver. However, the relationship between fatty acids and the expression of hepatic PPARγ during cirrhosis regression remains unknown. In this study, we explored the serum metabolic characteristics and expression of PPARγ in patients with histological response to treatment with entecavir.

**Methods:** Sixty patients with HBV-related cirrhosis were selected as the training cohort with thirty patients each in the regression (R) group and non-regression (NR) group based on their pathological changes after 48-week treatment with entecavir. Another 72 patients with HBV-related cirrhosis and treated with entecavir were collected as the validation cohort. All of the serum samples were tested using ultra-performance liquid chromatography coupled to tandem mass spectrometry. Data were processed through principal component analysis and orthogonal partial least square discriminant analysis. Hepatic PPARγ expression was observed using immunohistochemistry. The relationship between serum fatty acids and PPARγ was calculated using Pearson’s or Spearman’s correlation analysis.

**Results:** A total of 189 metabolites were identified and 13 differential metabolites were screened. Compared to the non-regression group, the serum level of fatty acids was higher in the R group. At baseline, the expression of PPARγ in hepatic stellate cells was positively correlated with adrenic acid (*r* ^2^ = 0.451, *p* = 0.046). The expression of PPARγ in both groups increased after treatment, and the expression of PPARγ in the R group was restored in HSCs much more than that in the NR group (*p* = 0.042). The adrenic acid and arachidonic acid (AA) in the R group also upgraded more than the NR group after treatment (*p* = 0.037 and 0.014).

**Conclusion:** Baseline serum differential metabolites, especially fatty acids, were identified in patients with HBV-related cirrhosis patients who achieved cirrhosis regression. Upregulation of adrenic acid and arachidonic acid in serum and re-expression of PPARγ in HSCs may play a crucial role in liver fibrosis improvement.

## Introduction

Liver cirrhosis, one of the most threatening diseases worldwide, may develop into hepatocellular carcinoma or other decompensation events. Viral infection, especially hepatitis B virus (HBV) infection, and alcohol abuse are common pathogenic factors in China and Asia (GBD, 2017 Cirrhosis Collaborators, 2020). Cirrhosis has been shown to be reversed when pathogenic factors are removed (Chang et al., 2010; D'Ambrosio et al., 2012). However, not all patients benefit from antiviral treatment, and there remain 42%–73% of patients with chronic hepatitis B (CHB) with fibrosis or cirrhosis who fail to obtain satisfactory therapeutic effects in terms of histological changes following 48–78 weeks of entecavir treatment (Schiff et al., 2008; Chang et al., 2010; Sun et al., 2017; Sun et al., 2018; Wu et al., 2018).

A previous study revealed that metabolic pathways related to carbohydrates, amino acids, and lipids changed were altered under conditions of liver fibrosis or cirrhosis among humans, mice, and rats (Chang and Yang, 2019). Another study reported that several metabilites such as plasma lactate, tyrosine, methionine and phosphatidylcholines may predict survival in patients with decompensated cirrhosis (McPhail et al., 2016). In HBV-related cirrhosis, serum bile acids are closely associated with pathological progression (Wang et al., 2016). Metabolomics analysis or certain serum metabolites can distinguish and predict liver cirrhosis and decompensated cirrhosis (Wang et al., 2012). Thus, the progression of liver fibrosis is closely related to metabolomics, while there are fewer reports on the changes in metabolomics during fibrosis regression.

The peroxisome proliferator-activated receptor (PPAR) family regulates downstream targeted pathways, such as TGF-β, MAPKs, and NF-κB p65 (Li J. et al., 2021), and it is involved in the pathological process of liver fibrosis. A previous study showed that intrahepatic expression of PPARγ was significantly reduced in patients with liver fibrosis and cirrhosis. Many studies have focused on the role of PPARγ in hepatic stellate cell (HSC) activation (Bae et al., 2010). In hepatic steatosis, PPARγ is also related to lipid metabolism in hepatocytes (Wang et al., 2020) and has significant anti-inflammatory properties (Chinetti et al., 2003). Indeed, He et al. demonstrated that PPARγ activation was necessary for docosahexaenoic acid (DHA) to reduce liver fibrosis (He et al., 2019). However, the expression of PPARγ in the liver tissues of patients with HBV-related cirrhosis remains unknown, especially during the regression of cirrhosis. Meanwhile, the relationship between PPARγ and serum metabolites, especially fatty acids, involved in the histological response of HBV cirrhosis requires further exploration.

Regarding patients with HBV-related cirrhosis who are treated by entecavir, it remains unclear why some people show a histological response, while others do not, and the mechanism of action. In this study, we conducted metabolomics and multiplex immunofluorescence staining to unravel the relationship between serum metabolites and PPARγ expression in patients who histologically responded to treatment.

## Methods

### Clinical studies

#### Study participants

Patients were recruited from the cohort between September 2014 to October 2018 in 20 hospitals from 12 provinces of China (Li ZX. et al., 2021) (Clinical Trial. gov: NCT 02241590). All of the patients participated in the biobank initiative. At each research visit, serum samples were collected and immediately stored at −80°C. Liver biopsy was performed twice before and after 48-week entecavir treatment at a dose of 0.5 mg once daily. This study was approved by the ethics committee of Shuguang Hospital (approval No. 2014–331-27–01) and other participating hospitals. All of the participants provided informed consent for the study.

#### Definition of fibrosis regression

In this study, the efficacy criteria for entecavir on liver histology were assessed through the change in liver fibrosis stage from before (pre-) to after (post-) therapy using the Ishak scoring system (Ishak et al., 1995). According to the pathological histological results of the liver biopsy before and after treatment, a decrease in the Ishak score of at least 1 point was considered to indicate fibrosis regression or non-regression. Three experienced pathologists, who were blinded to the clinical information of the liver biopsy sample, reached a consensus on the Ishak fibrosis score on each biopsy following discussion.

#### Inclusion criteria

The key inclusion criteria were as follows: 1) aged 18–60 years; 2) serologically proven active CHB infection based on documented history and detectable levels of HBV DNA >20 IU/mL; 3) Ishak fibrosis score of 5 or 6 before treatment; and 4) completed a 48-week treatment course, followed by liver biopsy.

#### Exclusion criteria

Patients were excluded if they had a history of hepatic decompensation, other chronic liver disease within treatment, or poor compliance to antiviral treatment. Patients with no liver biopsy available for evaluation before and after treatment were also excluded.

### Blood sample preparation and clinical parameter analysis

Overnight fasting venous blood samples were obtained using the conventional method before initiating antiviral therapy. Samples were centrifuged at 3,000 *g* for 15 min at room temperature. Serum was aliquoted and stored at −80°C for metabolomics analysis. The following clinical parameters were measured by a conventional method: platelet count (PLT), white blood cells (WBC), hemoglobin (HGB), blood glucose, aspartate aminotransferase (AST), alanine aminotransferase (ALT), γ-glutamyl transpeptidase (GGT), alkaline phosphatase (ALP), albumin, total bilirubin (TBIL), triglyceride (TG), total cholesterol (TC), low-density lipoprotein (LDL) cholesterol, high-density lipoprotein (HDL) cholesterol, creatinine (Cr), prothrombin time (PT), alpha fetoprotein (AFP), hepatitis B e antigen (HBeAg), and HBV DNA levels.

### UPLC-MS (ultra-performance liquid chromatography coupled to tandem mass spectrometry) analysis

#### Chemicals and reagents

All of the standards for the targeted metabolites were obtained from Sigma-Aldrich (St. Louis, MO, United States), Steraloids Inc. (Newport, RI, United States), and TRC Chemicals (Toronto, ON, Canada). All of the standards were accurately weighed and prepared in water, methanol, sodium hydroxide solution, or hydrochloric acid solution to obtain individual stock solutions at a concentration of 5.0 mg/mL. The appropriate amount of each stock solution was mixed to create stock calibration solutions.

Formic acid was of Optima grade and was obtained from Sigma-Aldrich (St. Louis, MO, United States). Methanol (Optima LC-MS), acetonitrile (Optima LC-MS), and isopropanol (Optima LC-MS) were purchased from Thermo Fisher Scientific (FairLawn, NJ, United States). Ultrapure water was produced using a Mill-Q Reference system equipped with a LC-MS Pak filter (Millipore, Billerica, MA, United States).

#### Experimental procedure

The method was optimized according to a previously reported protocol (Lan et al., 2016). Serum samples were thawed in an ice bath to diminish sample degradation. The serum samples were processed according to the following steps: 20 μL of serum was added to a 96-well plate, which was then transferred to an Eppendorf epMotion Workstation (Eppendorf Inc., Hamburg, Germany); 120 μL of ice cold methanol and partial internal standards were automatically added to each sample and vortexed vigorously for 5 min; the plate was centrifuged at 4,000 *g* for 30 min (Allegra X-15R, Beckman Coulter, Inc., Indianapolis, IN, United States) and then returned to the workstation; 30 μL of supernatant was transferred to a clean 96-well plate, and 20 μL of freshly prepared derivative reagent was added to each well; the plate was sealed and derivatization was performed at 30°C for 60 min; after derivatization, 330 μL of ice-cold 50% methanol solution was added to dilute the sample; the plate was stored at 20°C for 20 min, followed by centrifugation at 4,000 *g* at 4°C for 30 min; 135 μL of supernatant was transferred to a new 96-well plate with 10 μL of internal standards in each well; serial dilutions of derivatized stock standards were added to the left wells; and finally, the plate was sealed for LC-MS analysis.

An UPLC-MS system (ACQUITY UPLC-Xevo TQ-S, Waters Corp., Milford, MA, United States) was used to quantify all targeted metabolites. The optimized instrument settings are described in the Supplementary Material.

#### Multiplex immunohistochemistry staining

Multiplex immunohistochemistry staining (mIHC) was performed to analyze the expression of various factors in liver tissue. Deparaffinized and hydrated tissue sections were boiled in sodium citrate buffer (pH 6.0) for antigen retrieval. mIHC was performed using a four-color immunohistochemistry staining kit (PANOVUE, 10079100020) according to the manufacturer’s protocol. Next, α-SMA antibody (1:400 dilution, #19245, CST) was incubated overnight and washed with TBS. Subsequently, 30 μL of proportionally diluted tyramide signal amplification fluorescent solution (1:100) was incubated at room temperature for 20 min. Then, the PPARγ antibody (1:300 dilution, ab59256, Abcam) was also processed following the same conditions and steps. The stained slides were scanned and quantified using the HALO Highplex FL (Indica Labs; Albuquerque, NM) analysis module of Halo software (Erber et al., 2021).

#### Statistical analysis

The raw data files generated by UPLC-MS/MS were processed using MassLynx software (v4.1, Waters, Milford, MA, USA) to perform peak integration, calibration, and quantitation for each metabolite. The powerful package R Studio was used for statistical analyses. Statistical algorithms were adapted from the widely used statistical analysis software packages in R studio (http://cran.r-project.org/). Multidimensional statistics were based on the orthogonal partial least square discriminant analysis (OPLS-DA) model. The overall contribution of each variable to the model was described by VIP (variable import in the project), and the threshold was usually set to VIP >1. Univariate analysis was based on *p*-value obtained from the *t*-test or Mann–Whitney *U* test, and the fold change was used to filter variables before drawing a volcano plot.

We selected differentially expressed metabolites based on the criterion as absolute values of log_2_FC > 0 and *p* < 0.05.

Continuous variables are expressed as the mean ± standard deviation and as median (Q1, Q3) in cases where the data were abnormally distributed. Only observed values were used in the data analyses. Normally distributed data were analyzed by *t*-test, while those that were non-normally distributed were analyzed by non-parametric test using SAS 9.4. *p*-values <0.05 were considered to be statistically significant.

## Results

**FIGURE 1:**
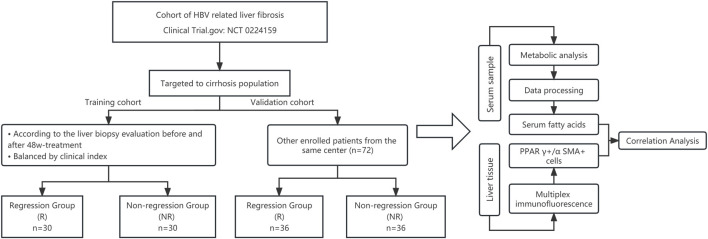
Flowchart of the study. The liver biopsy evaluation was based on the Ishak score.

The flowchart of this study is illustrated in Figure 1.

### Characterization of the study population

Considering the effects of sex, age, body mass index, and level of HBV-DNA on antiviral efficacy and metabolism (Sun et al., 2020), we balanced these factors to select cases for serum metabolomics tests. According to the pathological histological results of the liver biopsy, 60 patients were divided into regression and non-regression groups. The histology of the liver biopsies, as assessed by the Ishak scoring system, including HE and Sirius red staining, is shown in Supplementary Figure S1. The demographic and clinical characteristics of the two groups are detailed in Table 1. The two groups showed no significant differences in demographic and clinical characteristics before antiviral treatment (*p* > 0.05).

**TABLE 1:** Demographic and clinical characteristics of patients with liver fibrosis before antiviral therapy.

Group	Regression group (n = 30)	Non-regression group (n = 30)	Statistics	*p-*value
Male/female, n	20/10	20/10	—	—
Age (y)	43 ± 7.33	42.67 ± 9.65	0.151	0.248
BMI	23.54 ± 2.58	24.38 ± 2.95	−1.164	0.881
ALT (IU/L)	39 (26, 63.05)	40 (24.75, 60.35)	−0.126	0.9
AST (IU/L)	38.5 (29, 49.1)	50.45 ± 27	−0.547	0.584
Alb (g/L)	44.25 (41.03, 47)	41.5 (37.13, 45.23)	1.382	0.167
TBIL (μmol/L)	12.95 (9.25, 18.51)	13.35 (10.13, 18.2)	−0.266	0.79
GGT (IU/L)	77.9 ± 68.19	75.07 ± 52.78	0.18	0.858
ALP (IU/L)	100.17 ± 25.77	92.81 ± 33.9	0.918	0.363
Cr (μmol/L)	70.73 ± 16.03	70.8 ± 20.38	−0.014	0.989
WBC (×10^9^/L)	5.35 ± 1.8	4.66 ± 1.2	1.713	0.093
HGB (g/L)	141 (137, 151)	140 (131.5, 148.5)	−0.845	0.398
PLT (×10^9^/L)	128.37 ± 54.58	104.89 ± 35.73	1.763	0.06
PT (s)	12.53 ± 1.49	13.55 ± 1.69	−0.044	0.965
TC (mmol/L)	4.16 ± 0.94	4.15 ± 1.04	0.026	0.979
TG (mmol/L)	1.14 ± 0.45	0.94 (0.67, 1.16)	1.678	0.093
HDL-CH (mmol/L)	1.3 ± 0.38	1.42 ± 0.33	−1.292	0.201
LDL-CH (mmol/L)	2.56 ± 0.89	2.41 ± 0.95	0.62	0.538
Blood glucose (mmol/L)	5.16 ± 0.85	5.26 ± 7.9	−0.427	0.672
AFP (μg/L)	6.98 (3.48, 20.33)	8.48 (3.88, 22.56)	−0.118	0.906
Positive HBeAg, n, %	13 (43.33)	14 (46.67)	0.067	0.795
Log_10_ HBV DNA (IU/mL)	4.84 ± 2.17	4.46 ± 1.85	0.326	0.471
Ishak score			0	1
F5, n, %	14 (46.67)	14 (46.67)		
F6, n, %	16 (53.33)	16 (53.33)		

Mean values are provided with SD, and medians are provided with quartiles (Q1, Q3).

Annotations: BMI, body mass index; ALT, alanine aminotransferase; AST, aspartate aminotransferase; Alb, Albumin; TBIL, total bilirubin; GGT, γ-glutamyl transpeptidase; ALP, alkaline phosphatase; Cr, Creatinine; WBC, white blood cell; HGB, hemoglobin; PLT, platelet count; PT, prothrombin time; TC, total cholesterol; TG, triglyceride; HDL-CH, High-density lipoprotein cholesterol; LDL-CH, Low-density lipoprotein cholesterol; AFP, alpha fetoprotein.

### Serum metabolomics analysis and discovery of differential metabolites

In this study, we applied a quantitative targeted metabolomics profile. Quality control (QC) samples were applied to ensure the stability of the test. The principal component analysis (PCA) scores with QC samples are shown in Supplementary Figure S2A. QC samples gathering near the center of the score matrix projection showed that the sample analysis process was stable. Samples 2, 11, and 49 were far from the center and were identified as outliers and not included in the following analysis. A total of 189 metabolites were identified. Fatty acids (23.28%), amino acids (21.69%), organic acids (14.81%), carbohydrates (8.47%), and carnitine (7.41%) were the top five metabolites found in the two groups (Supplementary Figure S3A).

We applied multidimensional and single-dimensional statistical methods to screen for differential metabolites. In multidimensional statistics analysis, the distribution of regression and non-regression populations in the PCA score map showed no significant difference between each sample except for the outlier, as mentioned before (Supplementary Figure S3B). The PCA scores showed that the detected metabolites in the two groups were relatively similar in composition. Further OPLS-DA analysis showed that the metabolites of the two groups were partly distinguished. Moreover, 59 metabolites were selected when considering the contribution of VIP and correlation coefficients (the screening condition was defined as VIP >1, Supplementary Figure S3C).

**FIGURE 2:**
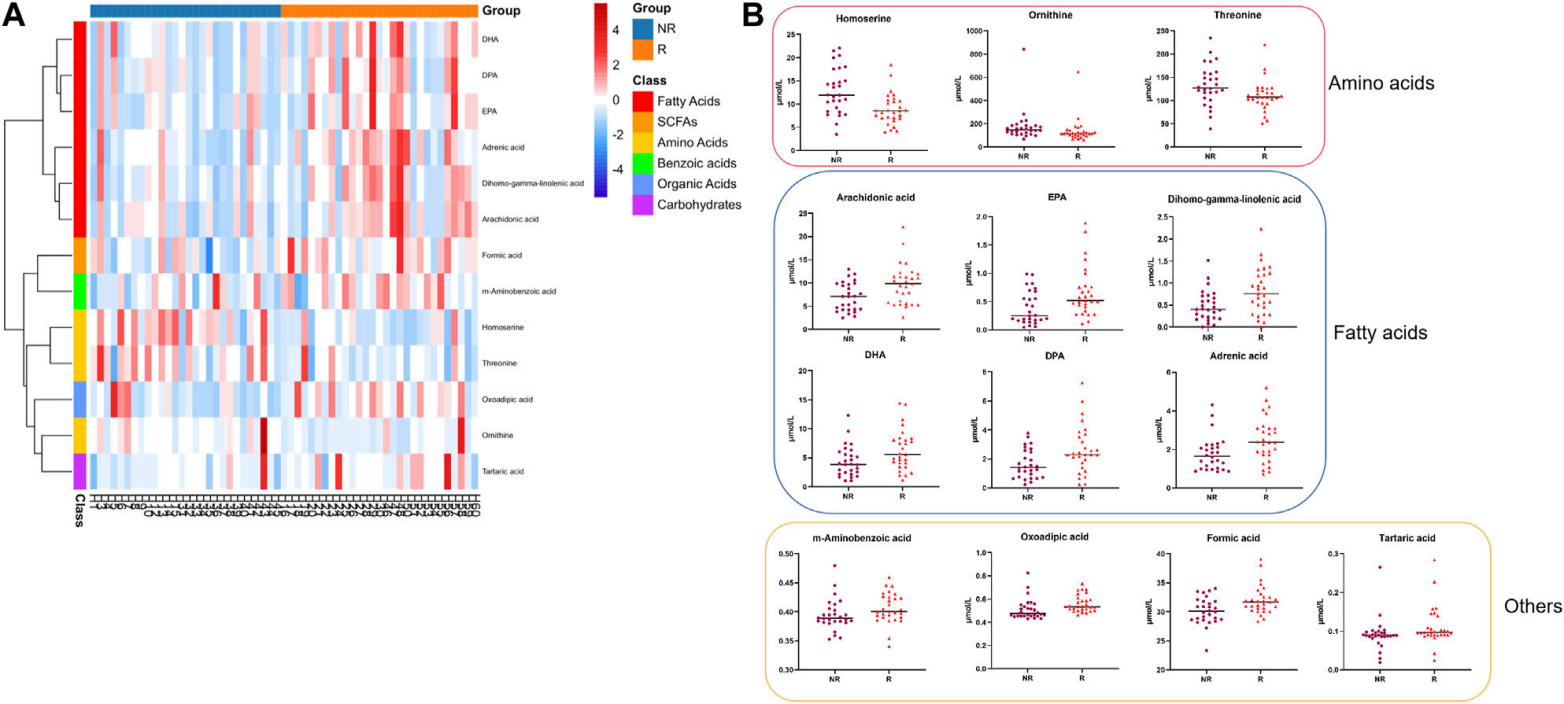
Differential serum metabolite expression between groups at baseline. **(A)**. Heatmap of differential metabolites. **(B)**. Serum level of 13 differential metabolites between the R and NR groups. R: Regression, NR: Non-regression.

We selected differential metabolites from univariate analysis according to the following criterion: *p* < 0.05 and the absolute value of log_2_FC (fold change) > 0. The volcano plot revealed 13 metabolites that were significantly different between the two populations (Figure 2).

Compared to the metabolic levels of the patients in the NR group, the levels of homoserine, ornithine, and threonine were lower in the patients in the R group. In contrast, the other 10 metabolites, including arachidonic acid and adrenic acid, were increased in the patients in the R group (Table 2).

**TABLE 2:** Identification of biomarkers for regression and non-regression groups.

No.	Metabolite	Class	HMDB	KEGG	R vs NR		
					log_2_ FC	VIP	*p*-value
1	Homoserine	Amino acids	HMDB0000719	C00263	−0.457	2.299	0.003
2	Oxoadipic acid	Organic acids	HMDB0000225	C00322	0.141	1.08	0.003
3	Ornithine	Amino acids	HMDB0000214	C00077	−0.427	0.754	0.011
4	Tartaric acid	Carbohydrates	HMDB0000956	C00898	0.122	1.029	0.011
5	m-Aminobenzoic acid	Benzoic acids	HMDB0001891	NA	0.042	1.179	0.016
6	Formic acid	SCFAs	HMDB0000142	C00058	0.051	1.362	0.019
7	Arachidonic acid	Fatty acids	HMDB0001043	C00219	0.5	1.589	0.022
8	Threonine	Amino acids	HMDB0000167	C00188	−0.214	1.457	0.028
9	EPA	Fatty acids	HMDB0001999	C06428	0.825	1.517	0.032
10	Dihomo-gamma-linolenic acid	Fatty acids	HMDB0002925	C03242	0.913	1.184	0.033
11	DHA	Fatty acids	HMDB0002183	C06429	0.511	1.34	0.041
12	DPA	Fatty acids	HMDB0006528	C16513	0.636	1.279	0.046
13	Adrenic acid	Fatty acids	HMDB0002226	C16527	−0.424	0.547	0.048

Annotations: FC, fold change; R, regression group; NR, Non-regression group; EPA, eicosapentaenoic acid; DHA, docosahexaenoic acid; DPA, docosapentaenoic acid.

### Identification and validation of serum fatty acids at baseline

The differential metabolites mainly belonged to fatty acids and amino acids, as shown in Figure 2. The serum levels of fatty acids, including adrenic acid, arachidonic acid, dihomo-gamma-linolenic acid, eicosapentaenoic acid (EPA), docosahexaenoic acid (DPA), and DHA, were higher in patients in the R group (all *p* < 0.05).

**FIGURE 3:**
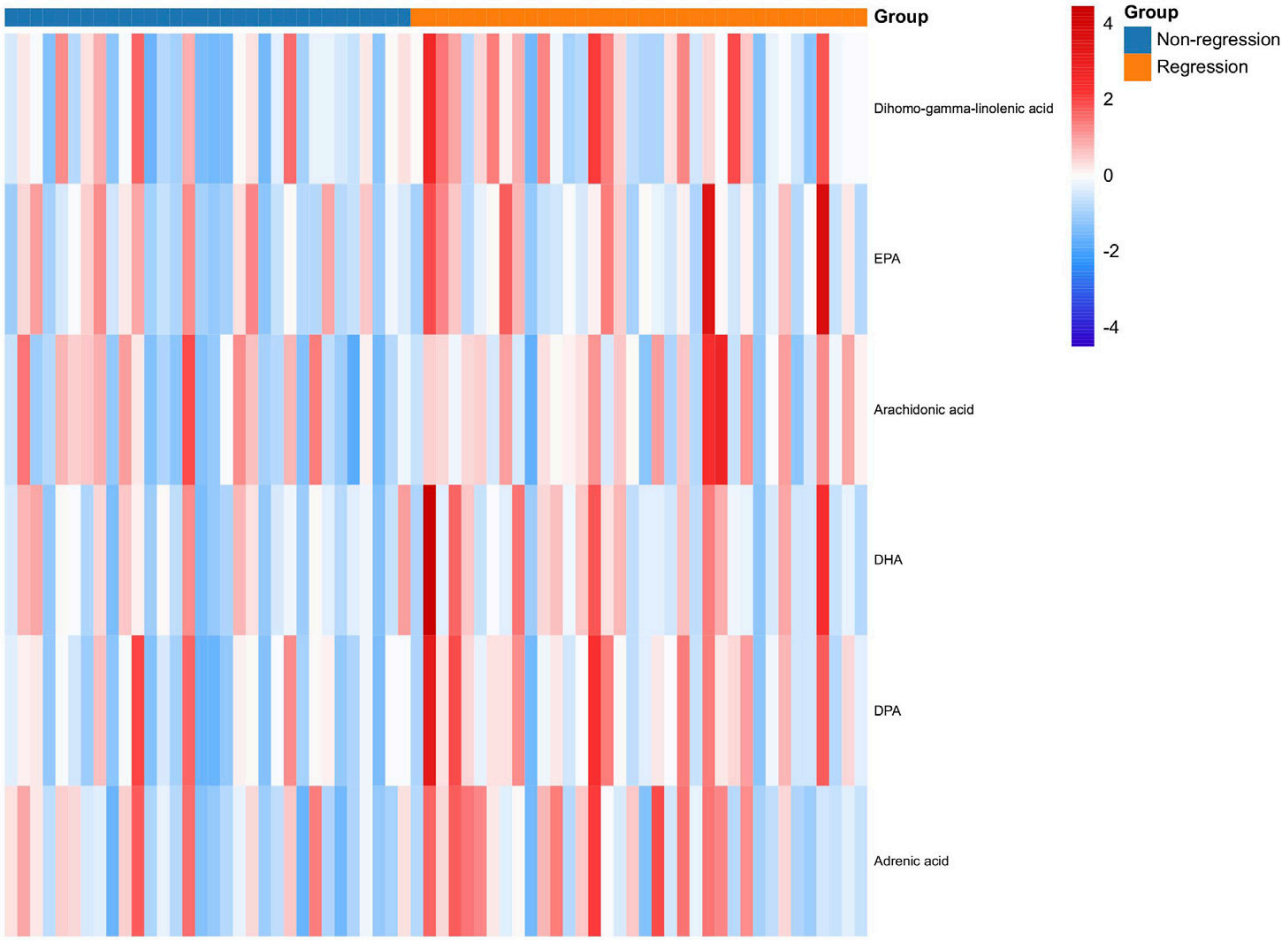
Expression of fatty acids between the R and NR groups in the validation cohort at baseline. R: Regression, NR: Non-regression.

To validate these fatty acids, the serums of other enrolled patients with HBV-related cirrhosis from the same centers were selected for metabolomics analysis. As shown in Table 3, there were no significant differences in sex, age, BMI, ALT, AST, ALB, TBIL, Cr, PT, WBC, HGB, PLT, AFP, and Ishak scores (*p* > 0.05), suggesting that the demographic and clinical data are comparable between the two groups. Meanwhile, the results in Figure 3 suggest that serum levels of fatty acids, including arachidonic acid, dihomo-gamma-linolenic acid, EPA, DPA, and DHA, were also higher in the patients in the R group (all *p* < 0.05).

**TABLE 3:** Demographic and clinical data of the validation cohort.

Validation patients	Regression group (n = 36)	Non-regression group (n = 36)	*p-*value
Male/female	22/14	22/14	*—*
Age (y)	43 ± 6.75	43 ± 8.75	0.894
BMI	23.75 ± 2.36	23.21 ± 2.62	0.397
ALT (IU/L)	40.74 ± 23.28	34.5 (25, 57.58)	0.702
AST (IU/L)	30.5 (24, 51.25)	36.50 (30, 53.13)	0.213
Alb (g/L)	43.03 ± 5.46	41.85 ± 6.15	0.695
TBIL (μmol/L)	13.43 ± 6.86	11.3 (8.13, 19.14)	0.724
Cr (μmol/L)	65.35 ± 16.95	68.25 ± 14.55	0.478
WBC (×10^9^/L)	4.62 (3.41, 6.04)	4.52 ± 1.63	0.444
HGB (g/L)	145 (126.5, 157.1)	139.44 ± 18.23	0.608
PLT (×10^9^/L)	121.83 ± 56.6	115.41 ± 57.15	0.665
PT (s)	13.32 ± 1.5	13.5 (12.4, 14)	0.568
TC (mmol/L)	4.09 ± 0.99	4.21 ± 1.05	0.659
TG (mmol/L)	1.05 (0.82, 1.29)	1.16 ± 0.49	0.534
HDL-CH (mmol/L)	1.38 ± 0.4	1.42 ± 0.41	0.735
LDL-CH (mmol/L)	2.39 ± 0.82	2.44 ± 0.79	0.808
Blood glucose (mmol/L)	5.01 ± 0.57	5.3 (4.56, 6.17)	0.09
AFP (ng/mL)	12.46 ± 13.22	23.70 ± 59.55	0.082
Positive HBeAg, n, %	19 (52.7%)	15 (41.5%)	0.345
Log_10_ HBV DNA (IU/mL)	4.35 ± 1.79	4.98 ± 2	0.197
Ishak score (n, %)			0.471
F5	23 (63.8%)	20 (55.6%)	
F6	13 (36.1%)	16 (44.4%)	

Annotations: BMI, body mass index; ALT, alanine aminotransferase; AST, aspartate aminotransferase; Alb, Albumin; TBIL, total bilirubin; GGT, γ-glutamyl transpeptidase; ALP, alkaline phosphatase; Cr, Creatinine; WBC, white blood cell; HGB, hemoglobin; PLT, platelet count; PT, prothrombin time; TC, total cholesterol; TG, triglyceride;HDL-CH, High-density lipoprotein cholesterol; LDL-CH, Low-density lipoprotein cholesterol; AFP, alpha fetoprotein.

### Correlations between serum fatty acids and expression of PPARγ in the liver

**FIGURE 4:**
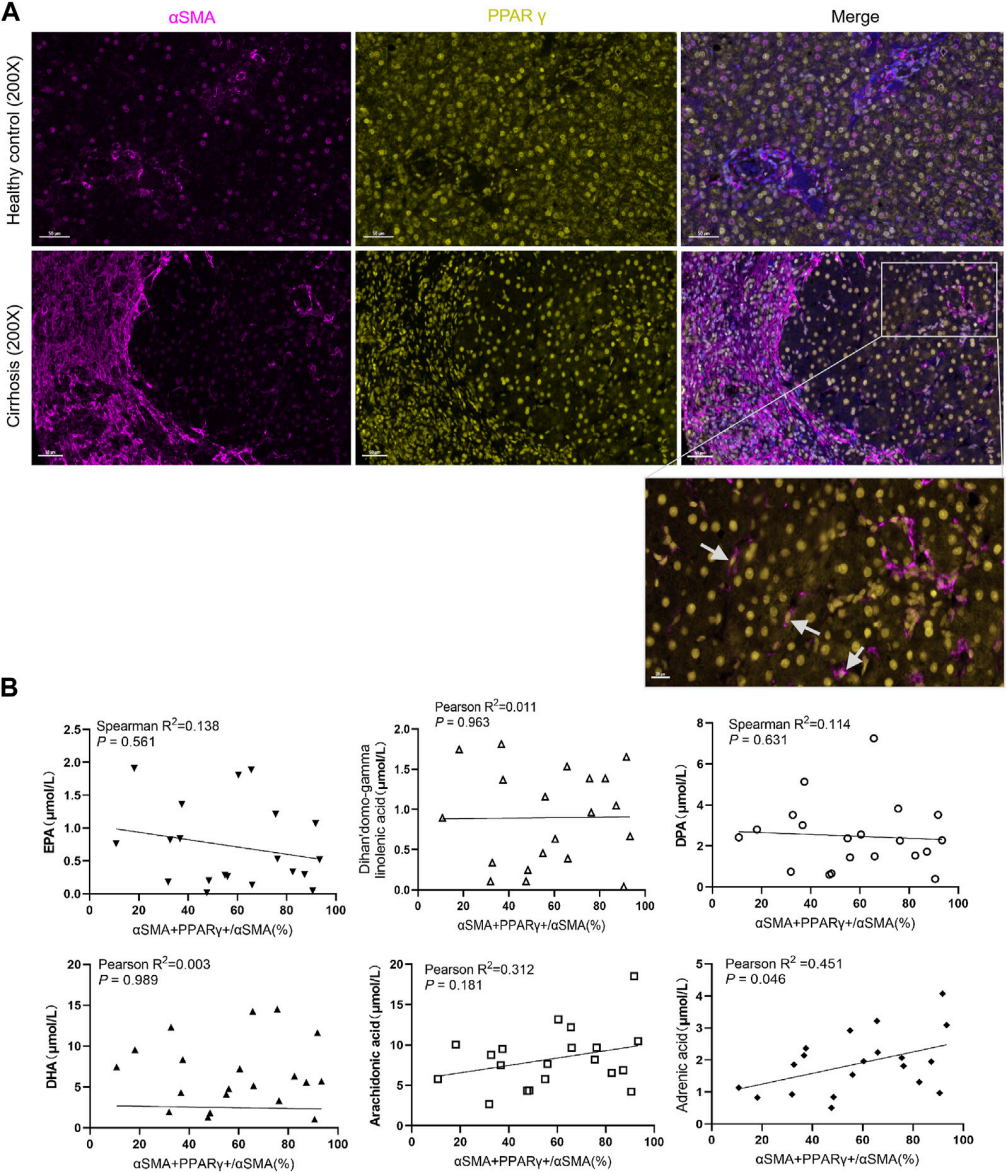
Expression of PPARγ in the liver and its relationship with serum fatty acids. **(A)**. Expression of PPARγ is downregulated in patients with chronic hepatitis B with cirrhosis. **(B)**. Correlation analysis between the proportion of PPARγ+αSMA+/αSMA + cells in the liver and the serum fatty acid level. The level of adrenic acid was positively correlated with the expression of PPARγ+ HSC.

PPARγ is mainly expressed in hepatocyte, HSCs, Kupffer cells, and liver sinusoidal endothelial cells (Boyer-Diaz et al., 2021). The general expression of PPAR in healthy liver was higher than that in the fibrotic or cirrhotic liver, especially fatty liver. We also observed that PPARγ was predominantly expressed in hepatocytes (Figure 4A). Previous studies have focused on the relationship between PPARγ loss and HSC activation (Liu et al., 2020). Interestingly, the serum level of adrenic acid was positively correlated with PPARγ^+^ HSCs in the cirrhotic liver (*r* ^2^ = 0.451, *p* = 0.046).

### Changes in serum fatty acid metabolism and PPARγ expression after treatment

**FIGURE 5:**
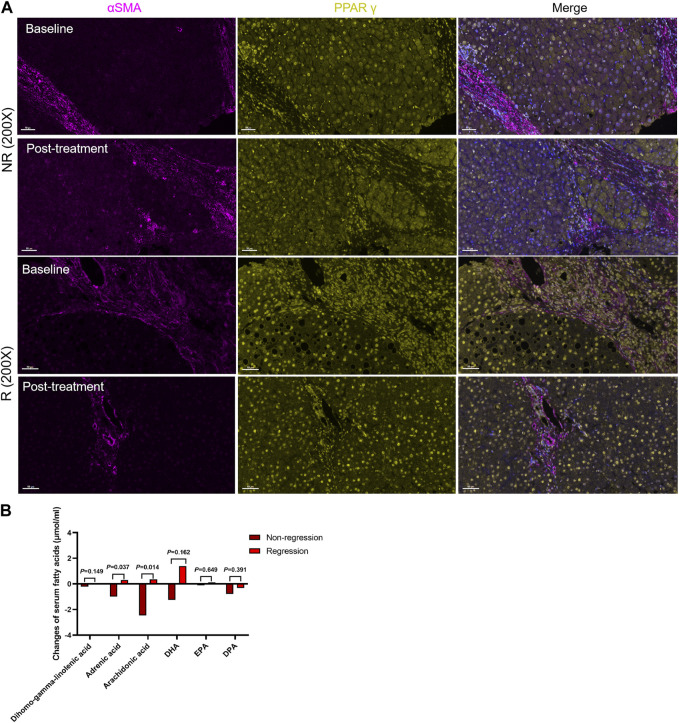
Changes in serum fatty acid metabolism and PPARγ expression after treatment. **(A)**. The R group expressed more PPARγ in hepatic stellate cells, while the proportion of PPARγ+ HSCs increased in the regression group following treatment. **(B)**. Changes in the levels of six serum fatty acids between the two groups.

After 48 weeks of antiviral treatment, PPARγ expression was restored in hepatocytes and HSCs in the regression group. Some PPARγ^+^ HSCs were seen at the collagen fiber deposits (Figure 5A). The changes in these six fatty acids stratified by liver fibrosis changes (fibrosis regression and non-regression) are shown in Figure 5B. Longitudinal change values of these six fatty acids were evaluated as predictors of fibrosis improvement at week 48, especially the change in adrenic acid and arachidonic acid (*p* = 0.037 and 0.014). Meanwhile, as shown in Table 4, the ratio of PPARγ^+^ in αSMA^+^ cells was elevated after treatment (0.63 ± 0.11 vs 0.87 ± 0.05, *p* = 0.042).

**TABLE 4:** Changes of PPARγ+cells in liver after antiviral treatment.

	Regression (n = 14)		*p*-value	Non-regression (n = 9)		*p*-value
	Baseline	Post-treatment		Baseline	Post-treatment	
PPARγ^+^ cells (%)	61.84 ± 11.15	85.17 ± 5.01	0.09	63.12 ± 7.48	80.99 (57.27, 88.45)	0.091
αSMA^+^ cells (%)	21.73 (17.75, 37.18)	11.53 ± 4.79	0.196	24.55 ± 4.59	26.48 ± 4.85	0.704
αSMA^+^PPARγ^+^ cells (%)	14.34 ± 4.6	10.57 ± 4.73	0.434	15.43 ± 3.33	20.23 ± 4.08	0.283
αSMA^+^PPARγ^+^/αSMA^+^ cells	0.63 ± 0.11	0.87 ± 0.05	0.042	0.63 ± 0.08	0.77 ± 0.06	0.079

## Dicussion

HBV-related liver fibrosis can regress or be reversed by antiviral therapy, such as entecavir or tenofovir. In previous studies, the regression rate following 78-week treatment ranged from 27% to 44% (Sun et al., 2017; Sun et al., 2018; Wu et al., 2018). However, it is still unclear whether the effect of antiviral therapy is affected by initial factors. It has been reported that metabolites can predict the pathological progression of CHB or the survival of decompensated liver cirrhosis (McPhail et al., 2016; Wang et al., 2016). Nevertheless, the relationship between baseline metabolites and the population with a histological response has not yet been reported.

Serum metabolomics is a method for diagnosing and treating diseases by detecting metabolites, intracellular substances, and microbial metabolites in the blood. In medicine, serum metabolomics play an important role in prevention, treatment, and prediction (Zhang et al., 2018; Ottka et al., 2021; Sun et al., 2021).

In this study, we found differences in serum metabolism between the regression and no-regression groups before antiviral treatment, which may influence the efficacy of treatment. Differential serum metabolites were mainly classified as fatty acids and amino acids and mainly included arachidonic acid, adrenic acid, EPA, and DHA. Fatty acids such as arachidonic acid paly a potential causal associations with the risk of NAFLD and cirrhosis (Chen et al., 2023). EPA is also involved in multiple pathways regulating hepatic inflammation and fibrosis (Fraser et al., 2022). The levels of EPA, arachidonic acid and other fatty acids also decreased in patients with HBV-related cirrhosis (Arain et al., 2017).

The level of fatty acid changes as liver fibrosis improves. Four eicosanoids, including adrenic acid, are significantly associated with the liver fibrosis stage at baseline in NAFLD (Caussy et al., 2020). Meanwhile, serum adrenic acid level was positively related to the expression of PPARγ^+^ αSMA^+^ in the cirrhotic liver. PPARγ is one of the marker of quiescent HSCs. Some studies have suggested that fatty acid metabolism affects the expression of PPARγ through regulating HSC activation (Liu et al., 2021; Ning et al., 2022). As is well known, upgrading eicosanoids, such as EPA or DHA, can inhibit HSC activation through re-expression of PPARγ (He et al., 2017). In this study, we observed that the expression of PPARγ^+^ αSMA^+^ in the livers of patients in the R group was higher and positively correlated with the serum level of adrenic acid at baseline.

Study showed that after 24-week treatment, changes in plasma eicosanoids, such as adrenic acid, arachidonic acid, DHA, and EPA, are associated with in liver fibrosis improvement in NAFLD (Caussy et al., 2020). Our findings showed a similar trend in HBV-related cirrhosis compared to nonalcoholic steatohepatitis. After 48 weeks of antiviral treatment, the serum levels of fatty acids, especially adrenic acid and AA, upgraded much higher in the R group, as the expression of PPARγ was increased in HSCs. This finding indicates that upregulation of adrenic acid and AA and PPARγ expressed in HSCs may play a crucial role in liver fibrosis improvement.

Our research has a few limitations that warrant discussion. First, we only included patients with HBV-related cirrhosis (Ishak F5-6). Therefore, our findings may be not applicable to patients with cirrhosis related to other factors. Second, the samples were from multicenter randomized double-blind trials in China. Although they covered a wide area, due to differences in living and eating habits, the findings remain to be verified by extending the study overseas. Finally, as this is an observational research, we need advanced assays to explore the potential mechanisms of metabolites and pathways; thus, a prospective study with larger samples and *in vitro* and *in vivo* experiments is required to verify the hypothesis.

## Conclusion

For HBV-related cirrhosis receiving entecavir treatment, patients who histologically respond to therapy had higher serum levels of several fatty acids at baseline. The expression of PPARγ was positively correlated with the serum level of adrenic acid. After treatment, a change in serum adrenic acid and arachidonic acid level were associated with fibrosis improvement, as the restoration of PPARγ in HSCs may be related to these two fatty acids. These findings provide potential therapeutic targets and pharmacological mechanisms of populations that respond to treatment.

## Data Availability

The original contributions presented in the study are included in the article/Supplementary Material, further inquiries can be directed to the corresponding authors.
