# Supplementary material for: Serum metabolomics characteristics and fatty-acid-related mechanism of cirrhosis with histological response in chronic hepatitis B
Source: Front Pharmacol. 2023 Dec 21;14:1329266. doi: 10.3389/fphar.2023.1329266 (PMC10764421; doi:10.3389/fphar.2023.1329266)
Supplement: Supplementary file 1 [file Presentation1.PDF]

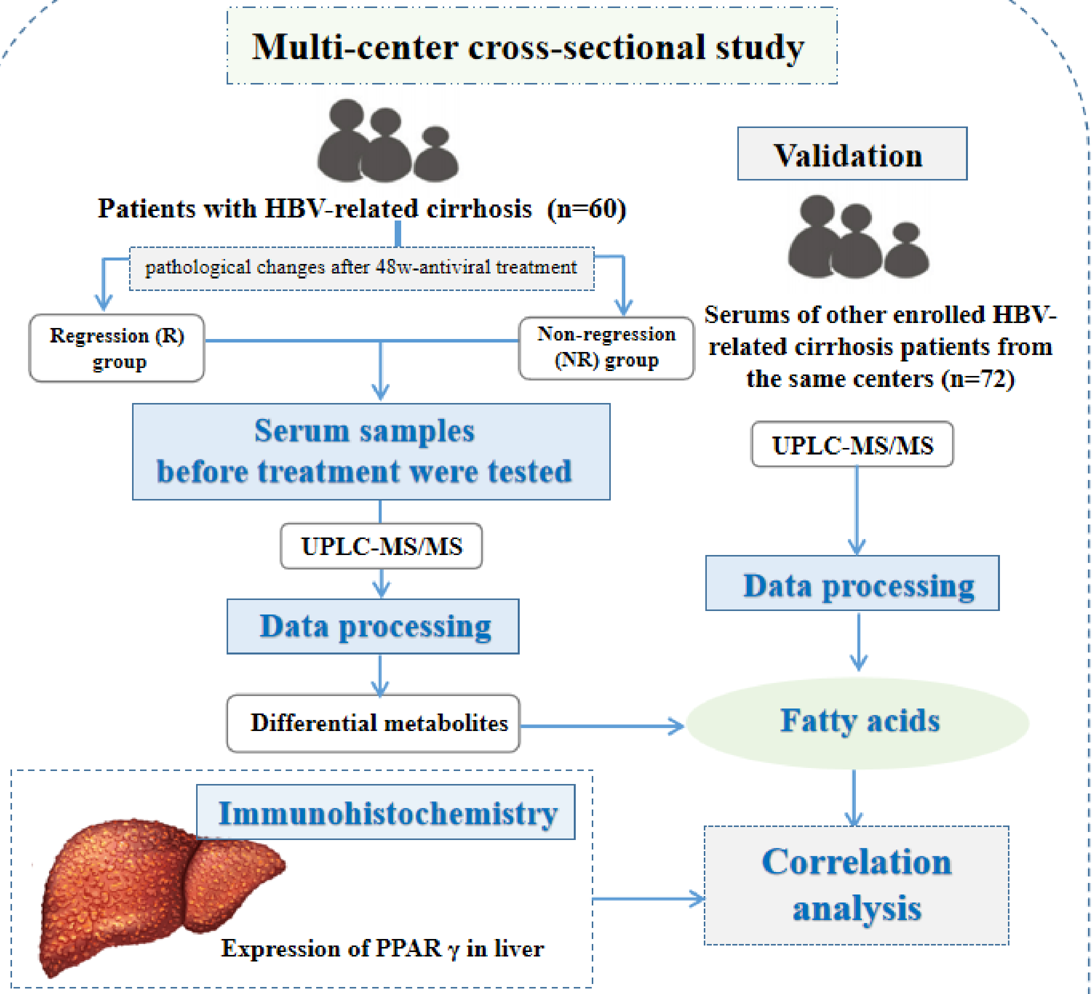

Highlights

1. Thirteen baseline serum differential metabolites, especially fatty acids, were identified in patients with HBV-related cirrhosis who achieved cirrhosis regression.
2. At baseline, the expression of PPARγ in HSCs was positively correlated with adrenic acid. The hepatic expression of PPARγ increased after treatment, and it was restored in HSCs much more in the R group.
3. The levels of adrenic acid and arachidonic acid in the R group also upgraded more than that in the NR group after treatment.
4. Upregulation of adrenic acid and arachidonic acid in serum with re-expression of PPARγ in HSCs may play a potential role in those achieved cirrhosis regression after antiviral treatment.
