# Supplementary material for: Serum metabolomics characteristics and fatty-acid-related mechanism of cirrhosis with histological response in chronic hepatitis B
Source: Front Pharmacol. 2023 Dec 21;14:1329266. doi: 10.3389/fphar.2023.1329266 (PMC10764421; doi:10.3389/fphar.2023.1329266)
Supplement: Supplementary file 2 [file DataSheet1.docx]

Supplementary Material

# Materials and Methods

## Chemicals and Reagents

All of the targeted metabolite standards were obtained from Sigma-Aldrich (St. Louis, MO, USA), Steraloids Inc. (Newport, RI, USA), or TRC Chemicals (Toronto, ON, Canada). All of the standards were accurately weighed and prepared in water, methanol, sodium hydroxide solution, or hydrochloric acid solution to obtain individual stock solutions at a concentration of 5.0 mg/mL. An appropriate amount of each stock solution was mixed to create stock calibration solutions.

Formic acid was of optima grade and was obtained from Sigma-Aldrich (St. Louis, MO, USA). Methanol (Optima LC-MS), acetonitrile (Optima LC-MS), and isopropanol (Optima LC-MS) were purchased from Thermo-Fisher Scientific (FairLawn, NJ, USA). Ultrapure water was produced by a Mill-Q Reference system equipped with a LC-MS Pak filter (Millipore, Billerica, MA, USA).

## Sample Preparation

Samples were thawed in an ice-bath to diminish sample degradation. Subsequently, 25 μL of plasma was added to a 96-well plate, before transferring to a Biomek 4000 workstation (Biomek 4000, Beckman Coulter, Inc., Brea, California, USA). Next, 120 μL ice cold methanol with partial internal standards was automatically added to each sample and vortexed vigorously for 5 min, before centrifuging the plate at 4000 g for 30 min (Allegra X-15R, Beckman Coulter, Inc., Indianapolis, IN, USA). Following centrifugation, the plate was returned back to the workstation, 30 μL of supernatant was transferred to a clean 96-well plate, and 20 μL of freshly prepared derivative reagent was added to each well. The plate was sealed and derivatization was conducted at 30°C for 60 min. After derivatization, 330 μL of ice-cold 50% methanol solution was added to dilute the sample, before storing the plate at –20°C for 20 min, and followed by centrifugation at 4000 g, 4°C for 30 min.

Subsequently, 135 μL of supernatant was transferred to a new 96-well plate with 10 μL internal standards in each well. Serial dilutions of derivatized stock standards were added to the left wells. Finally, the plate was sealed for LC-MS analysis.

## Instrumentation

An ultra-performance liquid chromatography coupled to tandem mass spectrometry (UPLC-MS/MS) system (ACQUITY UPLC-Xevo TQ-S, Waters Corp., Milford, MA, USA) was used to quantitate all targeted metabolites investigated in this study. The optimized instrument settings are briefly described below. The instrument performance optimization and routine maintenance were performed weekly. The UPLC-MS/MS instrument settings are shown in Table S1.

Table S1 UPLC-MS/MS instrument settings

| UPLC | |
| --- | --- |
| Column | |
| Column Temp. (°C) | ACQUITY UPLC BEH C18 1.7 µM VanGuard pre-column (2.1 × 5 mm) and ACQUITY UPLC BEH C18 1.7 µM  analytical column (2.1 × 100 mm) |
| Sample Manager Temp. (°C) | 40 |
| Mobile Phases | 10 |
| Gradient Conditions | A = water with 0.1% formic acid; and B = acetonitrile/IPA  (70:30) |
| Flow Rate (mL/min) | 0–1 min (5% B), 1–11 min (5%–78% B), 11–13.5 min (78%–95%  B), 13.5–14 min (95%–100% B), 14–16 min (100% B), 16–16.1  min (100%–5% B), 16.1–18 min (5% B) |
| Injection Vol. (µl) | 0.40 |
| MASS SPECTROMETER | 5.0 |
| Capillary (Kv) | |
| Source Temp. (°C) | 1.5 (ESI+), 2.0 (ESI–) |
| Desolvation Temp. (°C) | 150 |
|  | 550 |
| Desolvation Gas Flow  (L/Hr) | 1000 |

## Analytical Quality Control Procedures

The rapid turnover of many intracellular metabolites necessitates immediate metabolism quenching. The extraction solvents were stored in a –20°C freezer overnight, before adding to the samples immediately after thawing. We used an ice-salt bath to maintain the samples at a low temperature and to minimize sample degradation during sample preparation. All of the prepared samples were analyzed within 48 h of sample extraction and derivatization.

A comprehensive set of rigorous quality control/assurance procedures was employed to ensure a consistently high quality of analytical results, throughout controlling every step from sample receipt at laboratory to final deliverables. The ultimate goal of QA/QC is to provide reliable data for biomarker discovery study and/or to aid molecular biology research. To achieve this, three types of quality control samples, including test mixtures, internal standards, and pooled biological samples, are routinely used in our metabolomics platform. In addition to the quality controls, conditioning samples and solvent blank samples are required to obtain optimal instrument performance.

Test mixtures comprise a group of commercially available standards with a mass range across the system mass range used for the study samples. These samples were analyzed at the beginning and end of each batch run to ensure that the instruments were performing within laboratory specifications (retention time stability, chromatographic peak shape, and peak signal intensity). The retention time shift should be within 4 s, and the difference in peak intensity should be within 15% for LC-MS.

Internal standards were added to the test samples to monitor analytical variations during the entire sample preparation and analysis processes. The pooled QC samples were prepared by mixing aliquots of the study samples such that the pooled samples broadly represent the biological average of the whole sample set. The QC samples for this project were prepared with the test samples and injected at regular intervals (after every 14 test samples for LC-MS) throughout the analytical run.

Reagent blank samples are a mixture of solvents used for sample preparation and are commonly processed using the same procedures as the samples to be analyzed. The reagent blanks serve as a useful alert to systematic contamination. As the reagent blanks consist of high purity solvents and are analyzed using the same methods as the study samples, they were also used to wash the column and remove cumulative matrix effects throughout the study.

The calibrators consist of a blank sample (matrix sample processed without internal standard), a zero sample (matrix sample processed with internal standard), and a series of seven concentrations covering the expected range for the metabolites present in the specific biological samples. LLOQ and ULOQ are the lowest and highest concentrations of the standard curve that can be measured with acceptable accuracy and precision.

## Sample Run Order

To diminish analytical bias within the entire analytical process, the samples were analyzed in group pairs, but the groups were analyzed randomly. The QC samples, calibrators, and blank samples were analyzed across the entire sample set.

## Sample Control Procedure (ISO9001, QAIC/CN/170149)

Each received sample was accessioned into the Metabo-Profile LIMS system and was assigned by a unique LIMS identifier, which was associated with the original source identifier only and was used to track all of the sample handling, tasks, and results. The samples and aliquots were bar-coded and tracked by the LIMS system. All of the portions of any sample were automatically assigned their own unique identifiers by the LIMS when a new task was created; the relationship of these samples was also tracked. All of the samples were maintained at –80°C until processed. Metabo-Profile will help customers to store the remaining samples for up to 6 months before return or disposal. A formal disposal form authorized by the customer should be acquired.

## Data Control Procedure (ISO9001, QAIC/CN/170149)

The data retained on instrument control computers are immediately removed and transferred to a local data server (Metabo-Profile) for further data analysis located in a locked room. Metabo-Profile will not share any information with other customers until permitted. The data will be retained on the Metabo-Profile server for up to 6 months.

## Data Analysis

**Software**

The raw data files generated by UPLC-MS/MS were processed using the MassLynx software (v4.1, Waters, Milford, MA, USA) to perform peak integration, calibration, and quantitation for each metabolite. The powerful package R studio was used for statistical analyses.

# Quantitation

Mass spectrometry-based quantitative metabolomics refers to the determination of the concentration of a substance in an unknown sample by comparing the unknown to a set of standard samples of known concentration (i.e., calibration curve). The calibration curve is a plot of how the analytical signal changes with the concentration of the analyte (the substance to be measured). For most analyses a plot of instrument response vs. concentration will show a linear relationship, yielding a model described by the equation y = ax + b, where y is the instrument response e.g., peak height or area, a represents the slope/sensitivity, and b is a constant that describes the background. The analyte concentration (x) of unknown samples may be calculated from this equation.

# Statistics

Our proprietary software can perform a data collection, processing, interpretation, and visualization. Two types of statistical analysis are extensively performed in many metabolomics studies: 1) multivariate statistical analyses, including principal component analysis (PCA), partial least square discriminant analysis (PLS-DA), orthogonal partial least square discriminant analysis (OPLS-DA), and 2) univariate statistical analyses, including Student’s t-test, Mann–Whitney–Wilcoxon (U-test), ANOVA, and correlation analysis. Statistical algorithms are adapted from the widely used statistical analysis software packages in R studio [(http://](http://cran.r-project.org/))c[ran.r-project.org/).](http://cran.r-project.org/))


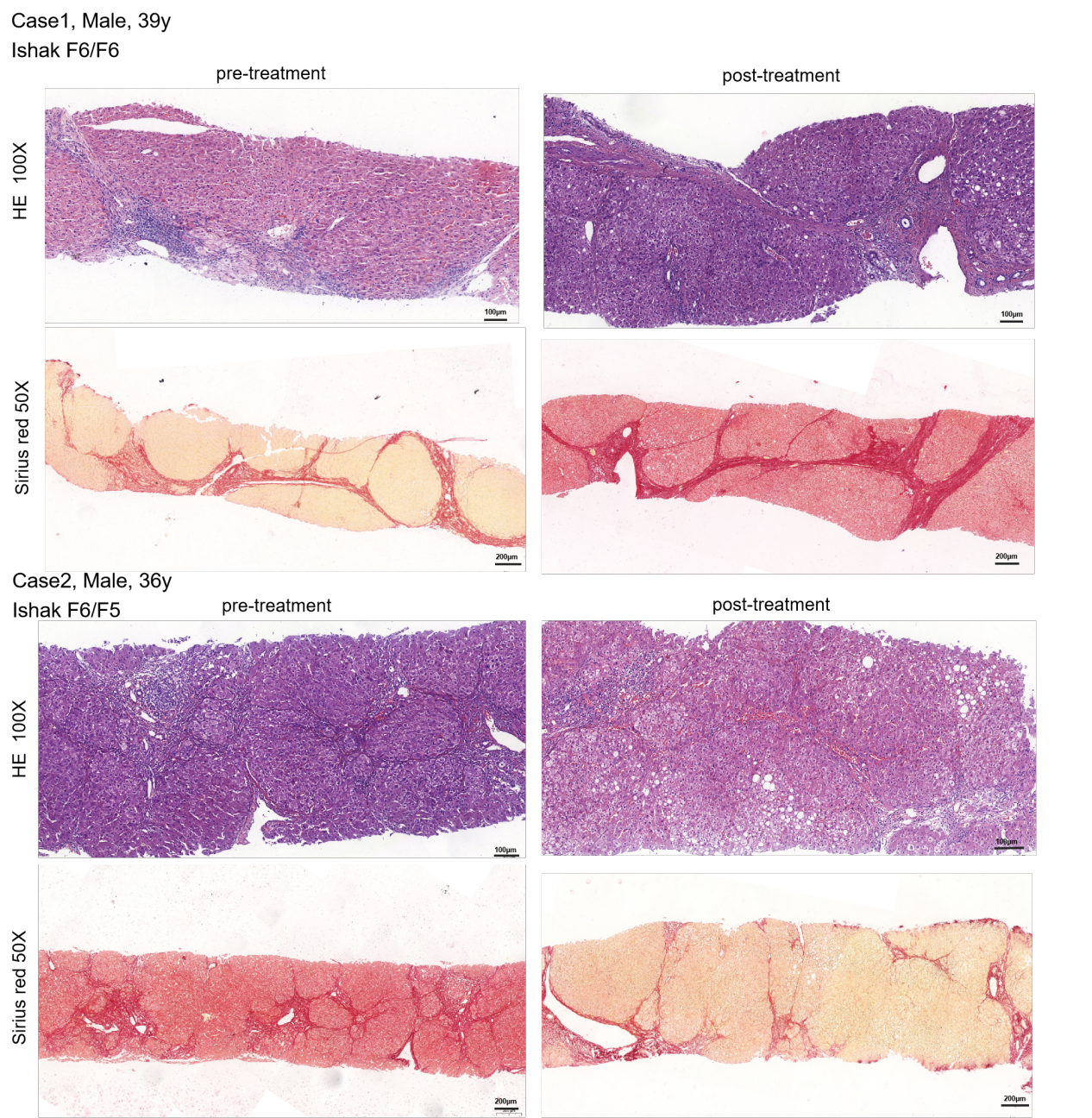


**Fig. S1. Liver biopsy samples of patients from the clinical cohort.** In case 1, the extent of liver fibrosis remained unchanged after 48-week antiviral treatment, whereas case 2 showed improvement according to the histology. HE: Hematoxylin-eosin staining.


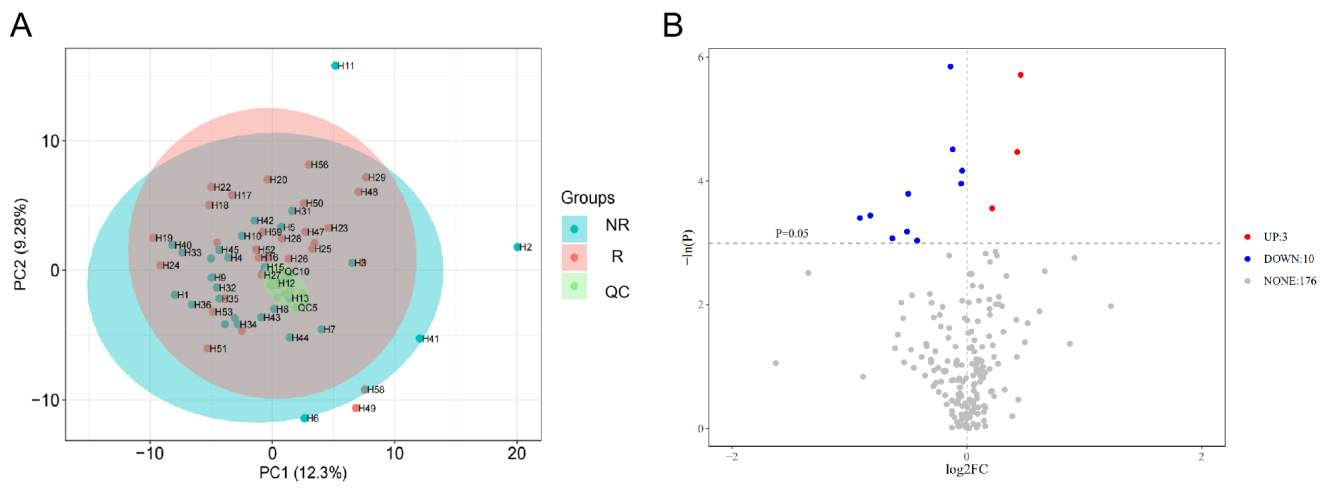


**Fig. S2. Serum metabolomics analysis and discovery of differential metabolites.**

1. PCA analysis of patients. B. Volcano plot based on OPLS-DA.

R: Regression group, NR: Non-regression group, PCA: Principal component analysis, OPLS-DA: Orthogonal partial least square discriminant analysis.


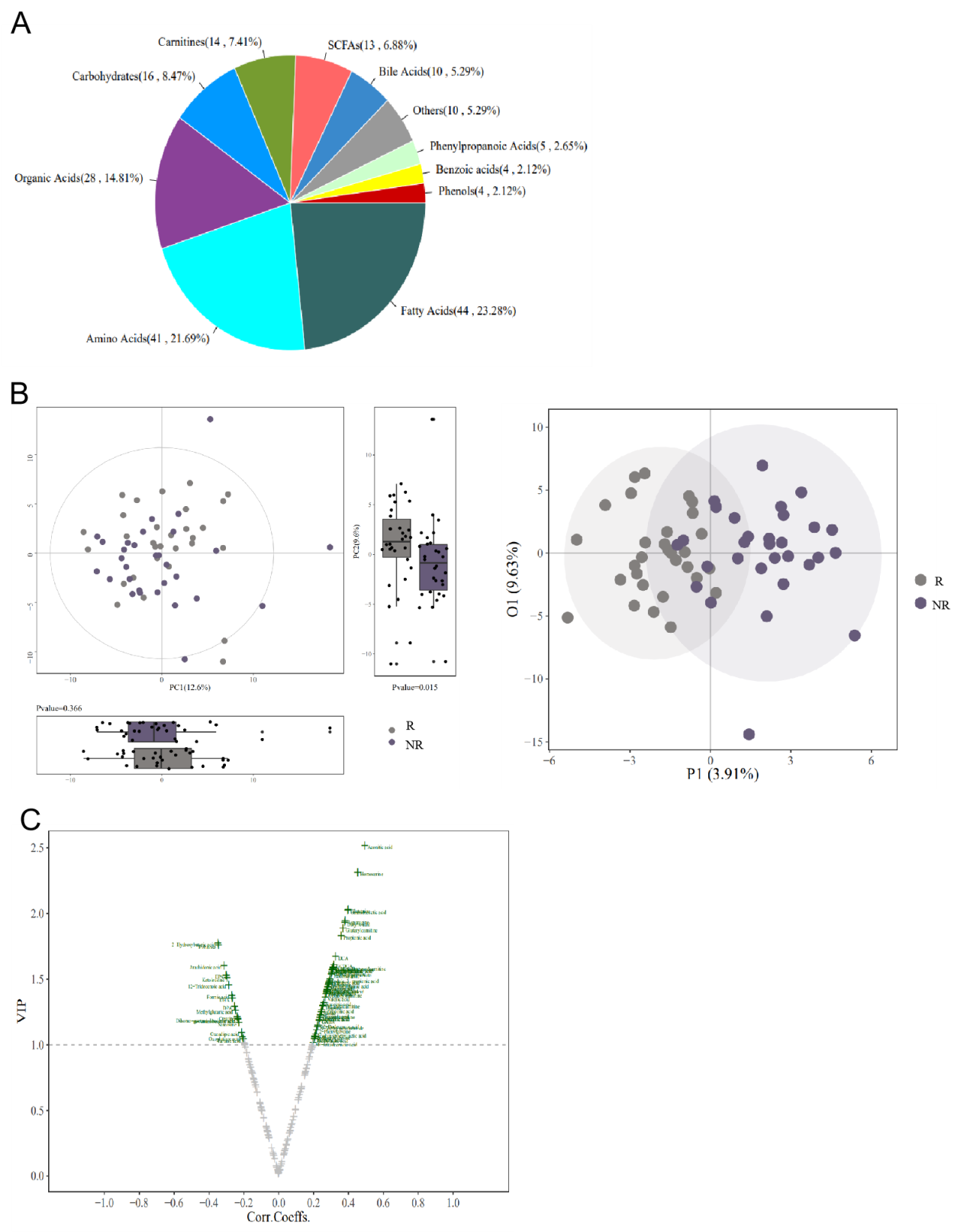


**Fig. S3. Differential metabolites selection.** A. Classification of identical metabolites. B: PCA score plot\OPLS-DA 2D score plot between the two groups. C. Volcano plot based on OPLS-DA.

R: Regression group, NR: Non-regression group, VIP:Variable import in the project, PCA: Principal component analysis, OPLS-DA: Orthogonal partial least square discriminant analysis.
